# Influence of Reduced Cortical Bone Compression by Implant Macrogeometry on Peri-Implant Bone Healing: An In Vitro and In Vivo Experimental Study

**DOI:** 10.3390/jfb17050217

**Published:** 2026-05-01

**Authors:** Sergio Alexandre Gehrke, Jaime Aramburú Junior, Tiago Luis Eilers Treichel, Antonio Scarano, Bruno Freitas Mello, Márcio de Carvalho Formiga, Sergio Rexhep Tari, Gustavo Coura, Gustavo Vicentis Oliveira Fernandes

**Affiliations:** 1 Department of Pharmaceutical Science, School of Health Sciences, Universidade do Vale do Itajai (UNIVALI), Itajai 88302-901, Brazilmarcio.formiga@bedentalschool.com.br (M.d.C.F.);; 2 Department of Implantology, Bioface/Postgrados en Odontologia/Universidad Catolica de Murcia, Montevideo 11100, Uruguay; 3 Department of Physiology and Pharmacology, Pro-Rectorate of Graduate Studies and Research (PRPGP), Universidade Federal de Santa Maria, Santa Maria 97105-900, Brazil; 4 Department of Surgery, Faculty of Medicine Veterinary, University of Rio Verde, Rio Verde 75901-970, Brazil; 5 Department of Innovative Technologies in Medicine & Dentistry, University of Chieti-Pescara, 66013 Chieti, Italy; ascarano24@gmail.com (A.S.);; 6 Department of Implantology, Be Dental School, Universidade do Vale do Itajai (UNIVALI), Saint Joseph 88102-700, Brazil; 7 Missouri School of Dentistry and Oral Health, A. T. Still University, St. Louis, MO 63104, USA; 8 GF10 Foundation, St. Louis, MO 63104, USA; 9 Centre for Interdisciplinary Research in Health (CIIS), Universidade Católica Portuguesa, 3504-505 Viseu, Portugal

**Keywords:** bone healing, bone-to-implant contact, cortical bone compression, dental implants, healing chambers, insertion torque, osseointegration, rabbit model

## Abstract

Background: Primary stability and long-term osseointegration depend on bone healing surrounding dental implants. Implant macrogeometry is crucial for controlling insertion torque and the biological reaction of peri-implant bone. This study assessed the impact of an implant design meant to lessen cortical bone compression on early bone healing. Methods: Forty titanium prototype implants (3 × 6 mm) were equally divided into Control (standard macrogeometry) and Test (macrogeometry with healing chambers) groups. Initial insertion torque was measured in vitro using synthetic bone blocks. Subsequently, an in vivo rabbit tibia model was used (*n* = 10 implants per group) to assess early healing. At 21 days, histological sections were analyzed for bone-to-implant contact (BIC%) at three cervical positions (C1, C2, and C3). Additionally, digital radiographs of the cervical region were evaluated using RGB color mapping, where distinct color channels quantified varying degrees of bone density. Results: The in vitro insertion torque for the Control group was significantly greater than the Test group (8.01 vs. 5.70 Ncm). The in vivo histomorphometric analysis indicated improved integration for the Test design, showing substantially higher BIC% at the C2 (59.30% vs. 40.30%) and C3 (42.10% vs. 17.90%) positions. Furthermore, radiographic RGB analysis revealed that the Test group possessed a higher blue channel contribution, indicating greater mineralized tissue density. Conclusions: These results imply that modifying implant macrogeometry to lower insertion torque and minimize cortical bone compression favorably enhances early cervical bone healing and osseointegration.

## 1. Introduction

The intricate process of bone healing surrounding dental implants relies on the integration of the implant surface with the surrounding bone tissue. This mechanism dictates both primary stability and long-term success [1,2,3,4,5]. While the biocompatibility of the implant surface is crucial, the macrogeometry of the implant, including its design, diameter, and length, as well as the surgical technique employed, strongly influences healing efficiency [6,7,8,9,10,11,12]. For many years, high insertion torque values were associated with successful osseointegration [13]. However, recent evidence demonstrates that surgical methods providing adequate osteotomy dimensions lead to faster and more efficient bone regeneration [14,15]. Consequently, novel implant designs have been developed to minimize bone tissue compression during placement [13,16,17,18,19,20,21,22].

To understand the rationale for these novel designs, one must evaluate how cortical and medullary bone respond differently to insertion trauma [16,17,18,23,24]. Cortical bone is denser and less vascularized than trabecular bone, making it far more vulnerable to mechanical stress and compression. During the placement of conventional implants, mechanical friction against the cortical bone is the primary means of achieving initial stability [25,26,27,28,29,30]. However, conventional cylindrical or conical implants can cause excessive bone compression. This is especially true in regions with dense cortical bone. Such compression may result in microfractures and delay bone healing [31,32,33]. Ultimately, this mechanical trauma can impede appropriate regeneration, postpone the production of high-quality bone, and raise the risk of peri-implant marginal bone resorption [34].

In this case, implant macrogeometry directly affects insertion trauma and the torque needed for fixation, in addition to the surgical approach used [16,17]. Conventional cylindrical or conical implants may cause excessive bone compression after implantation, especially in regions with dense cortical bone, which could result in microfractures and postpone bone healing [31]. Because cortical bone is less vascularized and denser than trabecular bone, it is more vulnerable to mechanical stress. Cortical bone compression or microfractures may impede appropriate regeneration, postpone the production of high-quality bone tissue, and raise the possibility of peri-implant bone resorption or failure [34].

Implants featuring healing chambers offer a biomechanical alternative to mitigate this stress. These designs can limit mechanical trauma and lower insertion torque. This maintains the integrity of cortical bone, particularly in the cervical region. Preserving this bone is essential for initial stability and peri-implant bone growth [13,16,17,24,31]. According to preclinical research, maintaining cortical bone during implantation encourages the development of thick, well-organized tissue, which minimizes early resorption and improves overall bone-implant contact [16,24,31]. Previous studies in rabbits comparing implants with and without healing chambers reported higher removal torque for the chambered implants, suggesting quicker and more efficient bone mineralization. Even though increased insertion torque does not automatically trigger crestal resorption, the macrogeometry design clearly directs the pattern and quality of new bone formation.

Experimental rabbit models are frequently employed to assess the biological impact of these implant macrogeometries. Rabbits exhibit a rapid rate of bone remodeling. Furthermore, this model allows the placement of multiple implants per animal, enabling reliable intra-animal comparisons between different designs [35,36].

Higher removal torque for implants with healing chambers was found in earlier research on rabbits comparing implants with and without healing chambers, suggesting quicker and more efficient bone mineralization when compared to conventional implants. These results imply that the pattern and quality of bone healing surrounding the implant are the primary differences, even though increased insertion torque does not always result in crestal bone resorption.

Based on this reasoning, the current study postulates that implants with healing chambers will favorably influence bone healing in the cervical region by reducing cortical compression during insertion. This should encourage increased bone density and superior bone-implant integration compared to conventional designs. Therefore, the purpose of this study was to assess differences in bone healing around titanium implants inserted with higher and lower compression (insertion torque) in rabbit tibiae, utilizing digital radiography and histological analyses with a specific focus on the cervical region.

## 2. Materials and Methods

### 2.1. Implants and Groups

**Figure 1:**
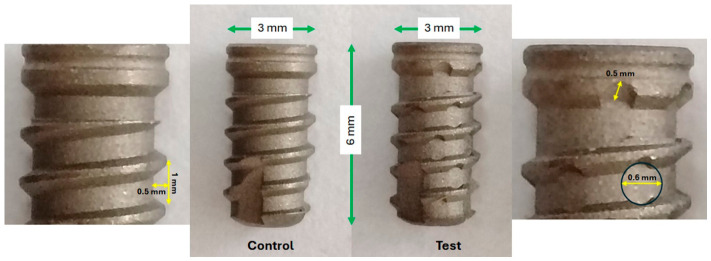
The two types of implants used in the study are shown schematically. The Control implant, which has a typical cylindrical shape without any alterations, is depicted in the left figure. The test implant, which has healing chambers with circular holes between the threads, is seen in the image on the right. The two implants are 6 mm long and 3 mm in diameter. Key aspects are measured in detail: both implants have a depth of 0.5 mm and a pitch of 1 mm between threads. The Test implant also has healing chambers with a diameter of 0.6 mm and a depth of 0.5 mm.

Forty commercially pure titanium (grade IV) cylindrical implants, each measuring 3 mm in diameter and 6 mm in length, were created for this investigation utilizing titanium oxide microparticles and sandblasting as a surface treatment (Implacil/Osstem, São Paulo, Brazil). There were 20 implants in each group; the Test Group had healing chambers, whereas the Control Group did not. Circular holes made between the implant’s threads defined the healing chambers. The two implant models and their dimensional properties are displayed in Figure 1.

### 2.2. In Vitro Insertion Torque Evaluation

**Figure 2:**
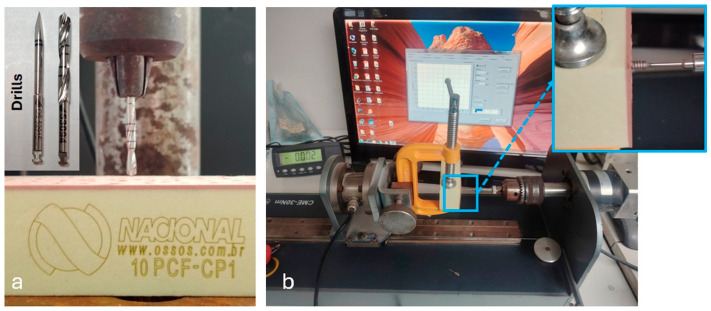
System for evaluating insertion torque in vitro. (**a**) A polyurethane foam synthetic bone block that mimics human bone is perforated using a lance-shaped drill and then a cylindrical drill (2.8 mm diameter, 6 mm depth). During the piercing process, a controlled speed of 800 rpm and 1 kg of pressure were used. (**b**) To ensure excellent measurement accuracy, a computerized digital torque meter was used to place the implants into the artificial bone. The measuring interface for torque monitoring during insertion is displayed on the computer’s screen.

For the in vitro insertion torque assessment, ten implants from each group were employed. This method was chosen since most surgical torque meters are analog and frequently inaccurate, making it challenging to acquire exact and trustworthy measurements during in vivo implantation. For this test, polyurethane foam synthetic bone blocks of 95 mm × 45 mm × 35 mm (Nacional Ossos, Jaú, Brazil) were utilized to simulate human bone. The American Society for Testing and Materials (ASTM) has accepted and acknowledged these materials as standard for testing bone instruments and implants [37]. A cortical thickness of 1 mm with a high density of 40 pounds per cubic foot (PCF) and a low-density medullary part of 10 PCF were employed to simulate the properties of rabbit tibial bone. A lance-shaped drill was first used to make the perforations, and then a cylindrical drill with a 2.8 mm diameter was used to reach a depth of 6 mm. A bench drill with a controlled speed of 800 rpm and a pressure of 1 kg was used to perform 20 similar operations (Figure 2a). This eliminated any eccentric motions that might happen while drilling manually in a clinical setting.

A computerized digital torque meter model CME-30 (Tecnica Industrial Oswaldo Filizola, São Paulo, Brazil) with an angular resolution of 0.002° was then used to insert the implants into the synthetic polyurethane bone block, reducing measurement errors and enhancing experimental precision (Figure 2b). Every implant was placed to the depth of the prepared perforation at the cortical bone level. For assessment and statistical analysis, the highest torque value attained during implant insertion was utilized.

### 2.3. Animal Model and Care

This study was initially examined and approved by the Animal Ethics Committee of the University of Rio Verde (Rio Verde, Brazil) under protocol number 04/20. All procedures strictly followed the ARRIVE guidelines and complied with Brazilian norms for animal experimentation, as recommended by the National Council for the Control of Animal Experimentation (CONCEA) resolution number 15 of 2013 [38]. Furthermore, the study design incorporated the 3Rs principles (Replacement, Reduction, Refinement) to ensure the ethical use of animals.

Following these guidelines, five adult New Zealand rabbits (Oryctolagus cuniculus), weighing between 3.5 and 4.0 kg, were obtained from the University of Rio Verde Animal Facility. The rabbit model is well-established and widely validated in the literature for this type of osseointegration study. Prior to the onset of the trial, the animals were dewormed and allowed to acclimate to the laboratory environment and human handling for 15 days. Throughout the acclimation period and the duration of the trial, each animal was housed individually in appropriate enclosures with ad libitum access to food and water.

To maximize statistical power while adhering to the ethical principle of Reduction (minimizing sample size), an intra-animal study design was employed. In this setup, both the Test and Control implants were placed in the tibiae of each rabbit, allowing the animals to serve as their own controls and reducing inter-subject biological variability.

### 2.4. Surgical and Medicinal Procedures

#### 2.4.1. Anesthesia and Pharmacological Protocol

The animals were initially anesthetized via an intramuscular injection of a ketamine-xylazine mixture, administered in a single syringe using 35 mg/kg of ketamine (Dopalen; Ceva Saúde Animal Ltd., Paulínia, Brazil) and 5 mg/kg of xylazine (Anasedan; Ceva Saúde Animal Ltd., Paulínia, Brazil). Local anesthesia was established by subcutaneous infiltration of 4% articaine with epinephrine 1:100,000 (DFL, Rio de Janeiro, Brazil) at the intended surgical incision site on each tibia. To provide transoperative analgesia, meloxicam at 0.2 mg/kg (Maxicam; Ouro Fino Saúde Animal Ltd., Cravinhos, Brazil) and morphine at 5 mg/kg (Dimorf; Cristália Produtos Químicos Farmacêuticos Ltd., São Paulo, Brazil) were administered subcutaneously. Additionally, prophylactic antibiotic therapy was given preoperatively via a subcutaneous dose of 84,000 IU/kg of Pentabiotic (Zoetis Indústria de Produtos Veterinários Ltd., Campinas, Brazil).

#### 2.4.2. Surgical Procedure

Following tibial shaving, local antisepsis was performed using povidone-iodine solution and 70% alcohol. A linear incision measuring approximately 5 cm in length was created in the proximodistal direction, starting close to the joint and extending along the medial surface of each tibia. The tissues were gently elevated to expose the bone, and osteotomies were performed using the same drilling protocol described in Section 2.2. Each animal received four implants in total (two in each tibia). To ensure an equal anatomical distribution between the proximal (P) and distal (D) regions of the tibia, the implants were positioned as follows: in the right tibia, the Test Group implant was placed in the P region and the Control Group implant in the D region; in the left tibia, this arrangement was inverted.

### 2.5. Post-Operative Care and Euthanasia

#### 2.5.1. Postoperative Care

Following the surgical procedures, the animals were housed in individual cages and continuously monitored until they fully recovered from anesthesia. For the first 24 h post-surgery, analgesia was maintained via subcutaneous morphine (5 mg/kg) administered every six hours. Subsequently, anti-inflammatory control was sustained using subcutaneous meloxicam (0.2 mg/kg) once daily for five consecutive days. Because the preoperatively administered Pentabiotic provides broad-spectrum antimicrobial coverage for up to seven days, no additional postoperative antibiotic doses were required.

#### 2.5.2. Euthanasia and Sample Retrieval

At 21 days post-implantation, all animals were euthanized in strict accordance with CONCEA standards (Normative Resolution No. 37/2018). Euthanasia was performed via an intravenous overdose (50 mg/kg) of 3% pentobarbital (Euthanyle, São Paulo, Brazil). Clinical death was verified by a licensed veterinarian, who confirmed the absence of respiratory and cardiac activity, lack of a perceptible pulse, absence of the corneal reflex, and the presence of pale mucous membranes.

### 2.6. Sample Collection and Handling

Every sample (*n* = 10 per group) was designated for histological examination. Following collection, the samples were immediately immersed in 10% buffered formalin for seven days. They were subsequently dehydrated in a graded series of ethanol solutions (50–100%) and embedded in historesin (Technovit 7200 VLC; Kulzer, Wehrheim, Germany).

Prior to sectioning, each intact bone block comprising a tibia was radiographed using a portable digital system (IriX-ray DX 3000; Dexcowin, Seoul, Republic of Korea) at a fixed distance of 15 cm. These initial radiographs were taken in the lateromedial (L-M) direction (i.e., with the X-ray beam passing horizontally from the lateral to the medial aspect of the tibia). Data collection and image acquisition were performed using an intraoral digital sensor (RVG First; Trophy, Toulouse, France) and Trophy Imaging software (version 7.0.19.3.d1).

**Figure 3:**
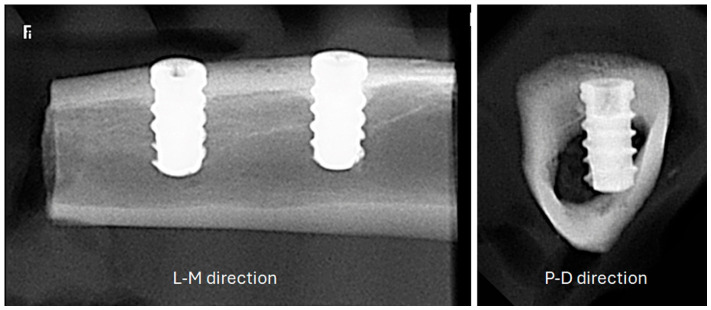
Digital radiographs that are representative of the bone samples in the two directions that were analyzed. The radiograph is displayed in the proximodistal (P-D) direction in the right image and in the lateromedial (L-M) direction in the left image.

Next, the tibial blocks were divided in half to isolate each implant in its own block. A second set of radiographs was then taken using the identical equipment and parameters, but in the proximodistal (P-D) direction (i.e., with the X-ray beam passing vertically along the long axis of the bone, from the proximal joint toward the distal extremity). Representative pictures of both radiographic directions are shown in Figure 3.

**Figure 4:**
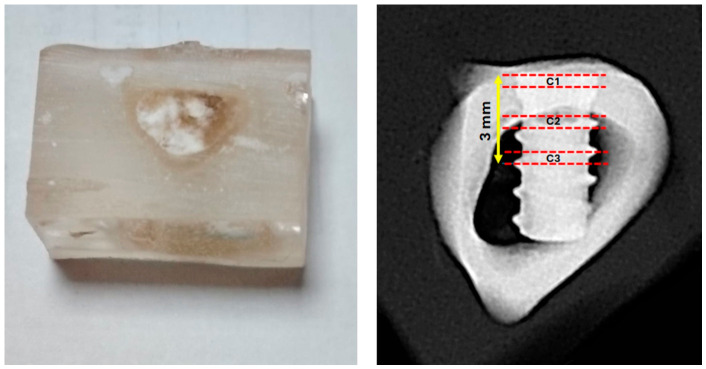
A unit sample is contained in a historesin block prior to sectioning. The three transverse incisions (C1, C2, and C3) within the first 3 mm of the implant were shown in the radiographic image taken in the lateromedial (L-M) direction, as shown by the red dashed lines, for further histological examination. An IsoMet 1000 machine was used to obtain the sections.

Following radiographic acquisition, transverse sections were obtained using an IsoMet 1000 machine (Buehler, Lake Bluff, IL, USA). As schematically depicted in Figure 4, three slices (designated C1, C2, and C3) were performed within the first three millimeters of the cervical region of each implant.

### 2.7. Histomorphometric and Histological Analysis

The slides were stained using the picrosirius hematoxylin method, and histological images were captured using a light microscope (E200; Nikon, Tokyo, Japan). ImageJ software (version 1.52v; National Institutes of Health, Bethesda, MD, USA) was utilized to calculate the bone-to-implant contact percentage (BIC%) for each designated transverse section level (C1, C2, and C3).

**Figure 5:**
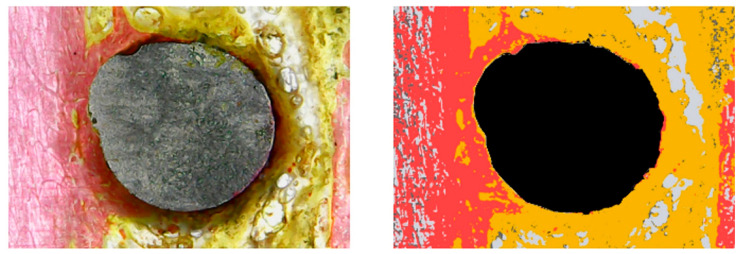
An illustration of the color-segmentation technique used in histomorphometric analysis. The segmented picture (**right**) and the original histology image (**left**). Bone tissue (BT), collagen tissue (CT), and marrow spaces (MS); MS: white/gray; CT: yellow/orange; BT: red/pink. The implant is represented by the black area, which was excluded from the histomorphometric analysis.

Furthermore, the area fractions of bone tissue (BT), collagen tissue (CT), and marrow spaces (MS) were quantified in each image within standardized rectangular regions of interest (4 × 6 mm). As depicted in Figure 5, these tissue percentages were calculated utilizing a digital color-segmentation method. Briefly, the ImageJ Color Threshold tool was applied to isolate specific hue ranges corresponding to the distinct stained tissues (red/pink = BT; yellow/orange = CT; and white/gray = MS). To ensure analytical accuracy and validate the digital segmentation, the computer-generated threshold areas were visually cross-verified against the original histological images by a calibrated examiner prior to the final area fraction computation.

For each part, a descriptive histological study was also carried out.

### 2.8. Radiographic Analysis

In accordance with our research group’s previously published methods [39], radiographs taken in both L-M and P-D directions were colorized using thermal pseudocoloring to depict differences in bone density in the RGB color space. Fundamentally, radiographic grayscale pixel intensity is directly proportional to the attenuation of X-ray photons by calcium and phosphate minerals; therefore, it serves as a tentative, non-invasive screening tool for actual bone mineralization [39]. By converting these continuous grayscale values into a discrete thermal RGB color scale, subtle variations in early peri-implant mineralization become more visually and quantitatively distinguishable. The following color interpretations were assigned using the Look-Up Table (LUT):

- Red indicates a lower density (less soft tissue or mineralized bone).
- Green denotes an intermediate density.
- Blue represents a higher density (more mineralized bone).

**Figure 6:**
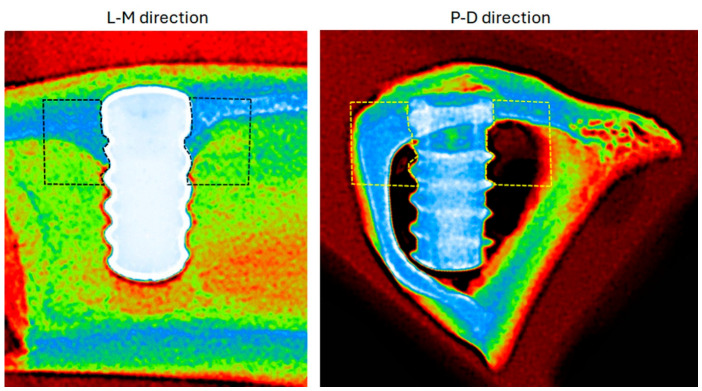
In order to depict changes in bone density in the RGB color space, radiographs taken in the lateromedial (L-M) and proximodistal (P-D) directions were colorized using thermal pseudocoloring. As shown by the dashed boxes, a region of interest (ROI) of 2 × 3 mm and situated lateral to each picture plant was defined in both image directions.

Next, a standardized 2 × 3 mm region of interest (ROI) was established lateral to each implant in both imaging directions (Figure 6). This specific ROI dimension and its lateral placement were strategically selected to encompass the immediate peri-implant bone-the critical zone for evaluating early bone remodeling and the biological response adjacent to the healing chambers-while deliberately excluding the highly radiopaque titanium implant body to prevent image artifacts from skewing the density data.

**Figure 7:**
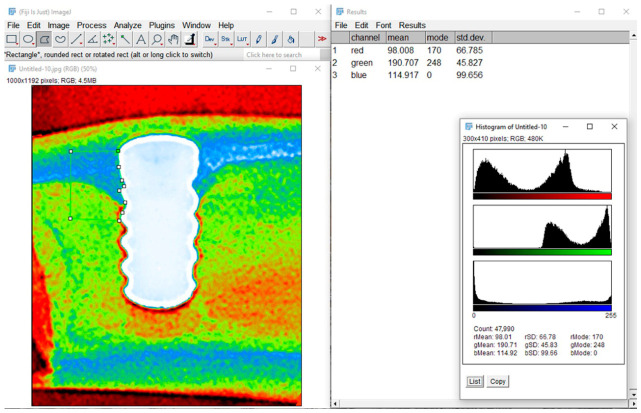
An illustration of RGB channel quantitative analysis using Fiji/ImageJ software (v. 2.9.0, NIH and LOCI, University of Wisconsin, Madison, WI, USA), inside a region of interest (ROI). The image with the chosen ROI is on the (**left**); the table with the mean intensity values for the red, green, and blue channels and the matching histograms produced by the Analyze > Color Histogram command is on the (**right**).

Fiji/ImageJ 2.9.0 (NIH and LOCI, University of Wisconsin, Madison, WI, USA) was used to quantitatively analyze the RGB channels. The Analyze > Color Histogram command was used to measure the presence (intensity) of each color (red, green, and blue), producing three distinct histogram graphs for each color component along with the mean value for each color. The relative color intensities inside the ROI are represented by the RGB intensity values derived from this study, which are expressed in arbitrary units. After the results were summed, the percentage contribution of each color within the ROI was computed (regarded as 100%). Evaluations and statistical analysis were conducted using these final percentage results (Figure 7).

### 2.9. Statistical Analysis

The relevant statistical software (GraphPad Prism, v.8) was used to examine the data. The Shapiro–Wilk test was used to determine the normality of the data, and parametric tests were used because a normal distribution was found.

Because an intra-animal design was utilized, observations within the same animal are inherently correlated. To adequately account for this correlation and isolate the implant effect from inter-animal variability, paired Student’s *t*-tests were used for all comparisons between the Control and Test groups (insertion torque, BIC%, and radiographic RGB channels). This pairing ensures that the statistical model controls for subject-level clustering.

Using paired Student’s *t*-tests, the percentage values of each color channel were compared between the Control and Test groups for the radiography analysis based on RGB color decomposition, separately for the lateromedial and proximodistal directions.

When applicable, the results are displayed as mean ± standard deviation and standard error. 5% (*p* < 0.05) was chosen as the significance level.

Cohen’s dz for paired comparisons was used to quantify the effect size, which showed significant differences between the Control and Test groups. A post hoc power analysis (α = 0.05) showed statistical power greater than 99% for the key outcomes, and the sample size was determined based on the experimental design and viability of the animal model.

While the small sample size (*n* = 5 rabbits) limits the overall statistical power, the intra-animal paired design maximizes the detection of intra-subject differences. A post hoc power analysis (α = 0.05) was conducted, and the sample size was determined based on the ethical principles of animal reduction (3Rs), experimental design, and viability of the animal model for exploratory studies.

## 3. Results

### 3.1. Torque Test Results

**Figure 8:**
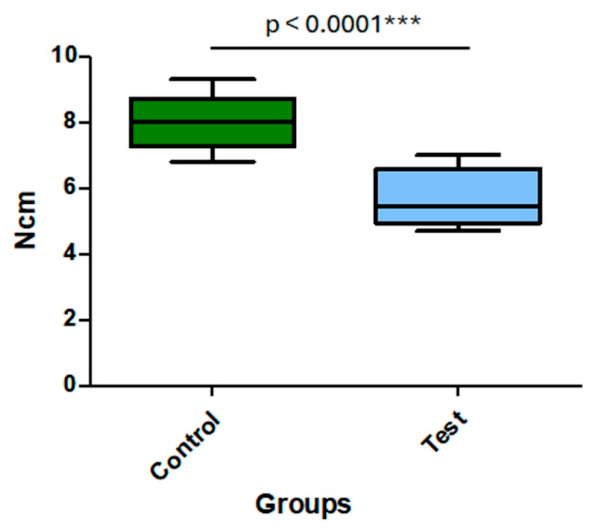
The distribution of insertion torque values (Ncm) obtained from the in vitro assay for implants in the Control and Test groups demonstrates the statistically significant difference between the groups.

A statistically significant difference between the groups was shown by the in vitro test findings comparing insertion torque between the two implant devices. The mean insertion torque was 8.01 ± 0.82 Ncm for the Control group and 5.70 ± 0.85 Ncm for the Test group (*p* < 0.0001). Figure 8 shows the distribution of individual values for both groups.

### 3.2. Histological Analysis Results

#### 3.2.1. Bone-Implant Contact

**Figure 9:**
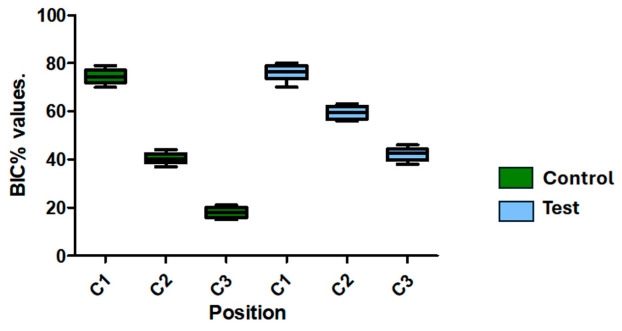
Bone-implant contact percentage (BIC%) values for the Control and Test groups at the three predetermined sites (C1, C2, and C3) are represented in a box plot. Whiskers show the lowest and maximum values, the middle line displays the median, and boxes show the inter-quartile range (Q1–Q3).

Table 1 and Figure 9 show the findings of the bone–implant contact percentage (BIC%) measurements at the three pre-selected locations (C1, C2, and C3). There were no statistically significant changes between the Control and Test groups at location C1. On the other hand, locations C2 and C3 showed statistically significant differences. At C2 (59.30% vs. 40.30%) and C3 (42.10% vs. 17.90%), the Test group’s mean BIC% values were much higher than those of the Control group; these differences were statistically significant (*p* = 0.0002).

#### 3.2.2. Bone Tissue, Collagen Tissue, and Medullary Spaces

Table 2 displays the results of the histomorphometric investigation. There were no discernible changes between the groups at the coronal area (C1). In comparison to the Control group, the Test group had less collagen and marrow space and more bone tissue in the more apical areas (C2 and C3). These results show that the test group’s apical regions had more bone growth.

#### 3.2.3. Descriptive Histological Analysis

**Figure 10:**
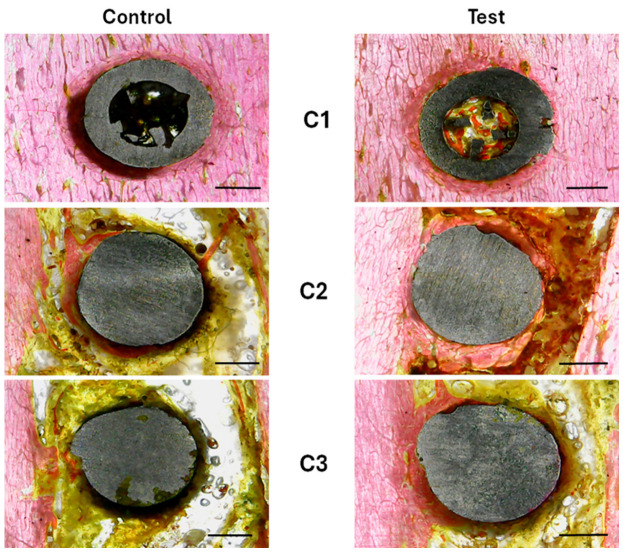
The Control and Test groups’ representative histology pictures at the three designated locations (C1, C2, and C3). Both groups exhibit comparable tissue–implant interface characteristics at C1, with the implant surface and surrounding tissue coming into close contact. Qualitative differences between the groups can be seen at C2 and C3, where the Control group displays a less uniform interface with interfacial gaps and connective tissue interposition, while the Test group displays a more continuous and homogeneous bone contact along the implant surface. Connective tissue is yellow, whereas bone tissue is pink. 1 mm is the scale bar.

Figure 10 displays representative histological sections from the Control and Test groups at the three predetermined locations (C1, C2, and C3). Both groups had a comparable histological appearance at position C1, which was defined by close contact between the implant surface and the surrounding tissue. There were no obvious gaps or discontinuities at the bone–implant contact in either group, and the peri-implant region was primarily populated by thick tissue that was closely fitted to the implant.

Qualitative differences between the groups became more apparent at point C2. There were interfacial voids surrounding the implant surface and areas with less tissue contact in the peri-implant region of the control group. The Test group, on the other hand, showed a greater degree of direct contact between the implant and the surrounding tissue, as well as a more continuous and uniform tissue contact along the implant perimeter with less obvious gaps.

The group differences were even more noticeable at location C3. The control specimens showed more interfacial gaps, a more uneven distribution of the surrounding material, and little contact between the implant and the surrounding tissue. On the other hand, the test group exhibited better adaptation, a more consistent distribution of tissue surrounding the implant, and a noticeably higher degree of contact along the implant surface.

Overall, the histological observations show that the Control and Test groups exhibited similar tissue behavior at the most coronal position (C1), whereas the Test group showed a gradually improved tissue–implant interface at the middle (C2) and apical (C3) positions, which were distinguished by greater continuity and homogeneity of contact when compared to the Control group.

### 3.3. Radiographic Analysis Results

**Figure 11:**
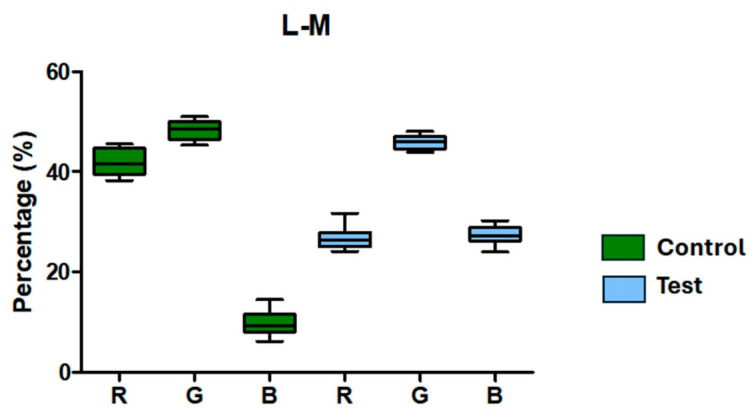
RGB color channel percentages for the Control (green) and Test (blue) groups obtained from radiography examinations in the lateromedial (L–M) direction. While the Test group exhibits lower R and higher G and B channel values, especially B, the Control group displays higher mean values in the R and G channels.

The RGB color decomposition-based radiography analysis results showed variations between the Control and Test groups in both assessed directions. In the lateromedial direction, the blue (B) channel had a lower mean (9.73%) while the red (R) and green (G) channels had larger means (41.96% and 48.32%, respectively) in the Control group. While the green (45.86%) and blue (27.36%) channels displayed greater values, especially the blue channel, the Test group’s mean red channel value (26.78%) was lower than that of the Control group. The information is shown in Figure 11 and Table 3. With comparatively small standard deviations and standard errors, measures of dispersion showed low variability in both groups, indicating measurement consistency.

**Figure 12:**
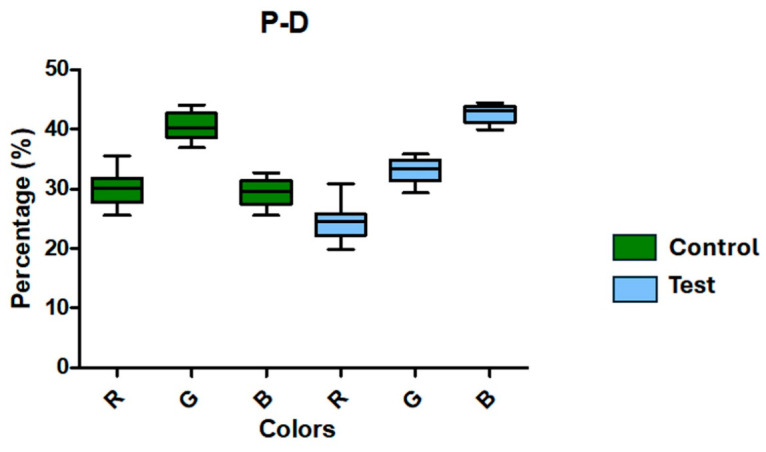
RGB color channel percentages for the Control (green) and Test (blue) groups derived from radiography analyses in the proximodistal (P–D) direction. While the Test group displays lower values on the red (R) and green (G) channels and a noticeable increase on the blue (B) channel, the Control group displays greater values on the green (G) channel and comparable values on the red (R) and blue (B) channels.

The Control group displayed rather uniform mean values for the red (30.10) and blue (29.39) channels in the proximodistal direction, with the green channel (40.51) exhibiting greater intensity. On the other hand, the Test group showed a significant increase in the blue channel (42.59) along with declines in the mean values of the red (24.32) and green (33.08) channels. The information is shown in Figure 12 and Table 4. Standard deviations and standard errors stayed low, suggesting a consistent data distribution, just like in the lateromedial analysis.

Overall, the RGB-based radiography analyses showed that the Control and Test groups’ chromatic intensity patterns differed, with the Test group’s blue channel contribution significantly increasing in both directions assessed.

## 4. Discussion

### 4.1. Study Aim and Main Findings

This study compared two models—one with healing chambers (Test group) and one without (Control group)—in order to assess bone healing surrounding cylindrical titanium dental implants. In comparison to conventional implants, implants containing healing chambers improved bone–implant integration, increased bone density, and promoted superior bone healing in the cervical area [13,16,17,24,31,40,41,42,43]. RGB decomposition radiographic analyses revealed a superior bone density pattern, especially in the blue channel, which is indicative of superior bone mineralization [39,44,45,46], and the Test group demonstrated a significant increase in bone–implant contact percentage (BIC%) at the more apical positions (C2 and C3) [16,18,24].

### 4.2. Differences in Insertion Torque and Mechanical Trauma

The Control group had higher torque values than the Test group, according to the insertion torque data, which showed a significant difference between the groups. However, because insertion torque was evaluated in synthetic polyurethane blocks to ensure standardized mechanical precision, the link between these in vitro mechanical findings and the subsequent in vivo biological healing remains indirect. In line with other research demonstrating that severe cortical bone compression during conventional implant insertion can cause microfractures and hinder bone regeneration, this suggests that implants with healing chambers can lessen mechanical trauma during implantation [13,16,17,31,34]. Reduced cortical bone compression, which may have maintained bone integrity in the cervical region and encouraged improved peri-implant bone growth, is probably what caused the lower insertion torque seen in the Test group [13,16,24]. This result is consistent with cortical bone’s known function in primary implant stability and its susceptibility to mechanical damage [16,17,18,23,24,25].

### 4.3. Influence of Macrogeometry on Bone Healing

The regional evaluation of bone-implant contact (C1, C2, and C3) was designed to reflect the distinct clinical demands placed on different segments of the implant. The coronal region (C1), corresponding to the cervical region, is clinically paramount to the long-term success of the implant. In this experimental model, the C1 region featured the same surface treatment (titanium oxide microparticles and sandblasting) as the rest of the implant and was placed in direct contact with the crestal cortical bone. Bone stability in this crestal zone dictates the architecture of the overlying peri-implant soft tissues, provides a biological seal against the oral environment, and resists the highest marginal stress concentrations during functional loading. Consequently, the equivalent performance of both groups at C1 indicates that the Test implant safely maintains crestal bone health. Therefore, the equivalence at C1 between groups confirms that the Test group does not compromise cortical stability, while the significant gains at C2 and C3 demonstrated its superior performance in promoting osseointegration within the challenging environment of trabecular bone. The middle (C2) and apical (C3) regions are primarily responsible for deep mechanical anchorage. The statistically significant improvements in BIC% and bone tissue formation observed in the Test group at C2 and C3 suggest a strong clinical advantage for achieving primary stability. Enhanced apical osseointegration is particularly relevant for modern clinical protocols, such as immediate implant placement in extraction sockets or installations in low-density bone, where apical engagement is heavily relied upon.

Significant differences in BIC% at locations C2 and C3 were found by histological investigation, with the Test group exhibiting significantly greater values than the Control group. These variations suggest that healing chambers may encourage more bone growth surrounding the implant, particularly in the cervical region. According to the research [16,17,18,23,24,31], the Control group, on the other hand, may have had more severe insertion trauma, potentially impairing bone repair, as seen by lower BIC% values in the more apical direction (C2 and C3 positions). Insertion trauma, bone remodeling, and early osseointegration are directly impacted by implant macrogeometry, which includes design, diameter, and the existence of healing chambers [6,7,16,17,31]. Importantly, while reduced cortical bone compression plays a key role, the improved bone healing cannot be definitively attributed to compression alone. Outcomes are closely associated with the implant’s surface topography and macrogeometry; the unique architecture of the healing chambers likely influences multiple biological factors, including clot stability, cell selection, haptotaxis, contact guidance, strain distribution, and the overall microenvironment that dictates bone ingrowth patterns.

These results were further corroborated by histomorphometric analysis. In comparison to the Control group, the Test group showed larger percentages of bone tissue (BT) and lower proportions of collagen tissue (CT) and marrow spaces (MS) in the more apical regions (C2 and C3), suggesting improved bone growth and more compact bone structure surrounding the implant. In contrast, the coronal region (C1), where bone tissue predominated in both groups, showed no discernible variations. These findings imply that the presence of healing chambers improves peri-implant tissue composition and promotes bone–implant contact, especially in areas more vulnerable to insertion-related damage.

### 4.4. RGB Radiographic Analysis and Implications for Healing

RGB-based radiography analysis revealed significant group differences. Used here as a tentative screening method rather than a confirmatory gold-standard analysis (such as micro-CT), the Test group displayed a significant increase in the blue channel in the lateromedial and proximodistal directions, suggesting increased bone density and mineralization surrounding the implant [17,24,31,39]. This probably results from better cortical bone preservation when implants with healing chambers are placed, which fosters the development of mineralized bone and effective osseointegration [13,16,17]. Particularly in cortical-rich areas, reduced cortical bone compression seems to positively regulate the bone healing pattern, supporting earlier research on the impact of implant design and insertion force on early bone response [14,15,23,24].

### 4.5. Concluding Remarks and Clinical Implications

Overall, the inclusion of healing chambers in the implant design promoted superior bone-implant integration and increased cervical bone density. By mitigating excessive cortical bone compression, a factor historically linked to poor regeneration and marginal bone resorption [13,16,17,18,31], this macrogeometric alteration yielded a more favorable biological response. Interestingly, although higher insertion torque values did not directly cause marginal bone loss in this study [13,47,48,49], the lower torque facilitated by the modified design still optimized the early bone-healing pattern. Despite the inherent anatomical limitations of the rabbit model, these findings suggest that minimizing cervical compression via implant macrodesign is a viable strategy to enhance early osseointegration. Further long-term clinical research is necessary to validate these benefits in human models.

## 5. Conclusions

Implants designed to lessen cortical bone compression showed reduced insertion torque and encouraged a more advantageous initial bone healing pattern in the cervical region. However, these findings represent strictly pre-clinical observations within the constraints of this in vitro and short-term (21 days) in vivo experimental study. In comparison to traditional implant designs, the existence of healing chambers was linked to greater bone mineralization and bone–implant contact, suggesting improved early osseointegration. While these results imply that implant macrogeometry plays a crucial role in regulating the early peri-implant bone response, immediate clinical applicability is limited. Further long-term studies are required to confirm whether these initial biological advantages translate into sustained clinical success.

## Figures and Tables

**Table 1:** For the Control and Test groups, the bone-implant contact percentage (BIC%) was assessed at three predefined locations (C1, C2, and C3). Mean ± SD is used to express the values (*n* = 10 per group). Statistically significant differences between groups at the same place are indicated by different letters (*p* < 0.05).

	Control			Test		
	C1	C2	C3	C1	C2	C3
Mean	74.50	40.30 ^a^	17.90 ^b^	76.00	59.30 ^a^	42.10 ^b^
Std. Deviation	2.799	2.214	2.132	3.266	2.541	2.726
Std. Error	0.8851	0.7000	0.6741	1.033	0.8035	0.8622

Std.: Standard; a and b: different indicate statistically significant differences between the Control and Test groups at the same position (*p* < 0.05).

**Table 2:** Histomorphometric analysis.

Positions	C1		C2		C3	
	Control	Test	Control	Test	Control	Test
BT	94.73 ± 0.99	94.80 ± 1.09	36.48 ± 1.88 ^a^	54.24 ± 1.67 ^ba^	15.40 ± 0.99 ^a^	35.48 ± 1.51 ^b^
CT	2.77 ± 0.72	2.90 ± 0.77	43.97 ± 2.51 ^a^	38.96 ± 2.13 ^b^	45.03 ± 1.28 ^a^	36.67 ± 4.46 ^b^
MS	2.40 ± 0.46	2.40 ± 0.66	19.55 ± 4.15 ^a^	6.80 ± 2.74 ^b^	39.57 ± 3.16 ^a^	27.85 ± 5.50 ^b^

BT = bone tissue; CT = Collagen tissue; MS = Medullary space. Different letters (a, b) indicate statistically significant differences between the Control and Test groups at the same position (*p* < 0.05).

**Table 3:** Radiographic analysis based on RGB color decomposition in the lateromedial direction for Control and Test groups. Values are expressed in percentage as mean, standard deviation and standard error.

	Control			Test		
	R	G	B	R	G	B
Mean	41.96	48.32	9.730	26.78	45.86	27.36
Std. Deviation	2.811	1.836	2.363	2.226	1.327	1.901
Std. Error	0.8888	0.5806	0.7472	0.7038	0.4196	0.6011

**Table 4:** Radiographic analysis based on RGB color decomposition in the proximal–distal direction for Control and Test groups. Values are expressed as mean ± SD.

	Control			Test		
	R	G	B	R	G	B
Mean	30.10	40.51	29.39	24.32	33.08	42.59
Std. Deviation	2.862	2.308	2.317	3.006	2.075	1.544
Std. Error	0.9050	0.7299	0.7328	0.9506	0.6560	0.4882

## Data Availability

The original contributions presented in the study are included in the article, further inquiries can be directed to the corresponding authors.
